# Nomogram Based on HRV for Predicting the Therapeutic Effects of Orthostatic Training in Children with Vasovagal Syncope

**DOI:** 10.3390/children11121467

**Published:** 2024-11-30

**Authors:** Xiaojuan Du, Ping Liu, Dandan Xiang, Chunyu Zhang, Junbao Du, Hongfang Jin, Ying Liao

**Affiliations:** 1 Department of Pediatrics, Peking University First Hospital, Beijing 100034, China; 18801238002@163.com (X.D.); pingliu@pku.edu.cn (P.L.); 2411110284@stu.pku.edu.cn (D.X.); chunyudoctor@pku.edu.cn (C.Z.); drjunbaodu@pku.edu.cn (J.D.); jinhongfang@bjmu.edu.cn (H.J.); 2 State Key Laboratory of Vascular Homeostasis and Remodeling, Peking University, Beijing 100191, China

**Keywords:** nomogram, orthostatic training, vasovagal syncope, prediction model, children

## Abstract

Background: This study intended to find out whether the parameters of heart rate variability (HRV) can predict the treatment efficacy of orthostatic training among pediatric cases of vasovagal syncope (VVS). Methods: Patients with VVS who underwent orthostatic training were retrospectively enrolled. Lasso and logistic regression were used to sift through variables and build the model, which is visualized using a nomogram. The model’s performance was evaluated through calibration plots, a receiver operating characteristic (ROC) curve, and decision curve analysis (DCA) for both datasets. Results: In total, 119 participants were included in the analysis, and 73 and 46 were assigned to the training and validation datasets, respectively. Five factors with nonzero coefficients were chosen based on lasso regression: age, the root means square of successive differences between normal sinus beats (rMSSD), standard deviation of the averages normal-to-normal intervals in all 5-min segments, minimum heart rate, and high frequency. Drawing from the logistic regression analysis results, the visual predictive model incorporated two variables, namely age and rMSSD. For the training dataset, the sensitivity was 0.686 and the specificity was 0.868 with an area under the curve (AUC) of 0.81 (95% CI, 0.71–0.91) for the ROC curve. For the validation dataset, the AUC of the ROC was 0.80 (95% CI, 0.66–0.93), while sensitivity and specificity were recorded at 0.625 and 0.909, respectively. In the calibration plots for both datasets, the predicted probabilities correlated well with the actual probabilities. According to the DCA, the visual predictive model gained a significant net benefit across a wide threshold range. Conclusions: Pediatric patients with VVS can benefit from orthostatic training using a visual predictive model comprising age and rMSSD.

## 1. Introduction

Vasovagal syncope (VVS), the main type of autonomic nerve–mediated syncope (NMS) in children, accounting for 70%–80% of children with syncope [1,2], can deteriorate an individual’s quality of life [3,4] and incur high health care costs [5]. Active intervention needs to be given to prevent syncopal episodes. Orthostatic training, a free and easy-to-practice measure, is a classic nonpharmacological therapy for patients with VVS. One possible explanation of the effects of orthostatic training in treating VVS is that it can favorably modulate the function of the autonomic system by balancing sympathetic–parasympathetic activities, which are considered to be crucial in the pathogenesis of VVS [6,7]. Di Girolamo et al. hypothesized that regular orthostatic training may desensitize the cardiopulmonary receptors considered to be responsible for neurocardiogenic responses [8]. However, according to previous studies, the efficiency of orthostatic training is unsatisfactory if chosen indiscriminately [9,10]. A study showed that 42.9% of patients aged from 16 to 68 years old had the recurrence of syncope and presyncope across a 16.9-month average follow-up [11]. Therefore, stratifying patients who may benefit from nonpharmacological interventions by some biomarkers may reduce unnecessary treatment and enhance individualized care for children with VVS.

Previous research in children with VVS suggests that electrocardiogram acceleration index, night-time diastolic blood pressure standard deviation, night-time diastolic blood pressure variation coefficient, and 24 h urine adrenaline may be biomarkers for selection of orthostatic training [12,13,14]. We wondered if there were data based on routine clinical examinations that could be new biomarkers.

Heart rate variability (HRV) indexes, the beat-to-beat variation in the cardiac cycle, originating from 24 h Holter monitoring, are easily obtained using non-invasive means. It can represent an individual’s cardiac autonomic status [15] and was regarded as an indirect biomarker of cardiac autonomic control [16,17]. The standard deviation of the average normal-to-normal (NN) intervals in all 5-min segments (SDANN) primarily reflect one’s circadian rhythms [18]. The root means square of successive differences between normal sinus beats (rMSSD) and the percent of successive NN intervals that differ by more than 50 ms (pNN50) are related to high-frequency power and parasympathetic activity [19], whereas the standard deviation of NN intervals (SDNN) is correlated with low-frequency power and signifies the balance between sympathetic and parasympathetic activity [20,21]. Low frequency (LF) is associated with sympathetic and parasympathetic nervous system modulation, while high frequency (HF) was defined by vagal modulation. The LF to HF proportion (LF/HF) reflects the overall balance of sympatho-vagal [22].

HRV indexes have been widely used as predictors of prognosis in conditions such as epilepsy [23], brain injury [24,25], liver failure [26], obsessive–compulsive disorder [27], psychotherapy [28], and depression [29]. And in the field of pediatric orthostatic intolerance, it was used to predict the incidence of syncope in children during Head-up tilt tests (HUTTs) [30] and the treatment effectiveness of metoprolol in pediatric postural orthostatic tachycardia syndrome [31], but the role of stratifying pediatric patients with VVS who receive orthostatic training has not been clarified. Orthostatic training can improve autonomic function, and it makes sense to predict the efficacy in advance [32]. Thus, here we investigated whether pediatric patients with VVS can benefit from a predictive model based on the parameters of HRV.

## 2. Materials and Methods

### 2.1. Study Design and Subjects

**Figure 1:**
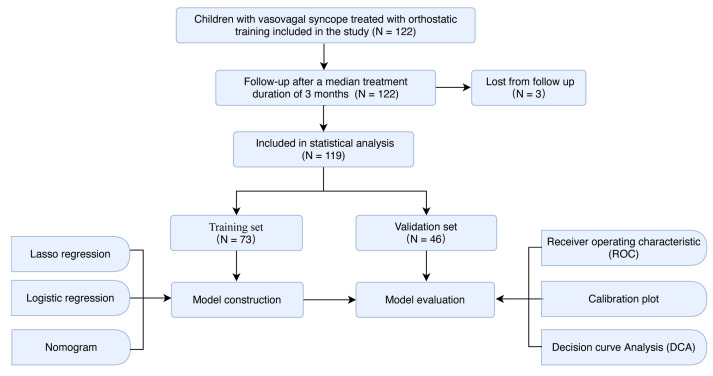
Workflow of the research performed to estimate the therapeutic effect of orthostatic training in children with vasovagal syncope.

The overall count of patients involved in this study was 122 (Figure 1); of them, 119 completed the study (60 males, 59 females; median age, 12 years [9,10,11,12,13]). The patient cohort was segmented into training and validation dataset groups based on their admission dates. Participants who were admitted before 28 December 2018 were placed in the training dataset, while those who were admitted after that day were placed in the validation dataset.

The diagnostic criteria for pediatric VVS [33]: (1) episodes of syncope; (2) syncope frequently triggered by predisposing circumstances, including prolonged periods of standing, rapid shifts in body position from sitting or squatting to standing, and exposure to hot and poorly ventilated environments; (3) a positive hemodynamic reaction on the head-up tilt test (HUTT); and (4) exclusion of alternative factors contributing to syncope-like episodes.

The following were the inclusion criteria: (1) pediatric patients of VVS who received orthostatic training between May 2009 and October 2022; and (2) treated with orthostatic training. The exclusion criteria were as follows: (1) no syncope attacks prior to admission for 3 months; (2) other causes of syncope, such as cardiogenic syncope; (3) taking other medicines or lacking data; and (4) treatment duration < 1 month.

This study obtained approval from the Institutional Ethics Committee of Peking University First Hospital (protocol code 2022496; approval date 22 February 2023 ), and permission was obtained from individual participants and their parents.

### 2.2. Head-Up Tilt Test

The HUTT was carried out within a controlled setting featuring subdued illumination, a warm temperature, and limited background noise. The patients were asked to fast for 4 h prior to the HUTT. Furthermore, it is imperative for patients to cease using medications that influence the autonomic nervous system of a timeframe corresponding to five times its half-life decay. The subjects were observed for 20 min while assuming a supine position on a table (SHUT-100A, Standard, Jiangyin, Jiangsu, China; and ST-711, Juchi, Beijing, China). At a 60° tilt, a table was used for the experiment, which lasted until a positive outcome was noted or until 45 min had been completed.

### 2.3. 24 h Holter Monitoring and Analysis of HRV Indexes

Twenty-four-hour Holter monitors (Mortara, Milwaukee, WI, USA) were used to evaluate HRV. Participants underwent 24 h Holter monitoring prior to orthostatic training, and they were instructed to abstain from vigorous physical activity, technological devices, and emotional arousal. The sampling rate was set at 10,000 Hz, with a frequency response spanning from 0.05 to 60 Hz. Analysis of HRV was conducted using an analyzer (H-Scribe 7.0; Mortara, Milwaukee, WI, USA). The values of the time-domain indexes for 24 h were acquired. The parameters collected from the Holter monitor were as follows: minimum HR during the entire day (minHR), maximal HR during the entire day (maxHR), mean HR during the entire day (mean HR), pNN50, rMSSD, SDANN, SDNN, standard deviation of the NN interval index (SDNN Index), LF, HF, and LF/HF.

### 2.4. Measurement Indicators

Demographic data (sex, age, and body mass index), heart rate (HR), systolic blood pressure (SBP), diastolic blood pressure (DBP), and parameters of HRV were collected using a Digital Medical Recording System (Kaihua, Beijing, China).

### 2.5. Treatment Protocol and Follow-Up

The orthostatic training procedure was as follows [12]. The participants were instructed to assume a standing position adjacent to a vertical surface with their heels positioned 15 cm away from the base of the wall. They were then directed to lean their upper back against the wall and maintain this position without moving. The time of this activity was gradually increased from 3 to 30 min depending on the individual’s orthostatic tolerance. The median period of treatment was 3 (3, 3) months. The therapeutic effect was appraised by a specialist, either via phone calls or outpatient visits after starting treatment. Three (2.5%) patients were lost to follow-up. No recurrence of syncope during follow-up was considered to be an effective response [12].

### 2.6. Data Analysis

Normality was assessed using the Kolmogorov–Smirnov test. Frequencies and percentages were used to express categorical data tested with the χ2 test. Normally distributed variables presented as the mean ± standard deviation were analyzed with Student’s t-test, while non-normally distributed variables presented as median (interquartile range) were analyzed with a Mann–Whitney U test. In the training dataset, all 18 covariates were selected with lasso regression to simplify the model, and the optimal penalty strength was selected by cross-validation, at which point the variables with non-zero coefficients were screened out and then were analyzed with logistic regression using the “step” function with “forward” correction to find the relationship between predictor variables and therapeutic efficacy. Multiple collinearities were detected using the statistical variance inflation factor (VIF). The Spearman correlation matrix is shown to indicate whether there are correlations within the variables selected or not. A visual predictive model was developed using a nomogram using the predicted variables based on logistic regression. The model’s performance was evaluated through calibration plots, ROC, and DCA. The dataset was processed using IBM SPSS Statistics 26 and R software (version 4.2.3) with the glm, rms, modEvA, MASS, cars, and rmda packages. A *p*-value threshold of 0.05 defined statistical significance.

## 3. Results

### 3.1. Variable Description of Included Participants

A total of 73 patients (median age, 11 years [9,10,11,12,13]; 37 [51%] female) were included in the training dataset (Figure 1). Of them, the orthostatic training was effective in 35 (47.9%). A total of 46 patients (median age, 12 years [9,10,11,12,13,14]; 22 [48%] female) were included in the validation dataset. Of them, orthostatic training was effective in 24 (52.2%), while it was ineffective in 22 (47.8%). The indicators involved in the prediction model did not exhibit significant intergroup differences (Table 1).

In the training dataset, sex (*p* = 0.903), body mass index (*p* = 0.947), total syncope attacks before treatment (*p* = 0.523), therapy duration (*p* = 0.585), SBP (*p* = 0.623), DBP (*p* = 0.246), and maxHR (*p* = 0.855) did not differ significantly between the effective and ineffective subgroups (Table 2). In contrast, age, SDNN Index, SDNN, rMSSD, SDANN, pNN50, LF, and HF were higher in the effective versus ineffective group (*p* all <0.05), whereas minHR, mean HR, and LF/HF were significantly lower in the effective compared to the ineffective group (*p* all <0.05).

### 3.2. Model Variable Screening

**Figure 2:**
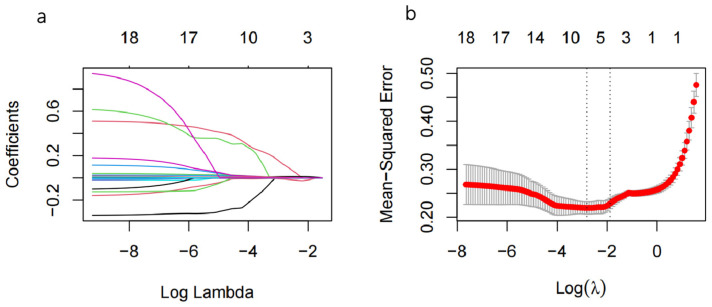
Factors chosen in the lasso regression for estimating the therapeutic effect of orthostatic training in children with VVS. (**a**) Lasso coefficient profile plot. The regression coefficient of each independent variables varied as λ changed. The color lines stand for different variables. As λ increased, the model exhibited a higher compression degree. (**b**) Cross-validation plot for the penalty term. The vertical line on the left corresponds to the λ value that yields the minimum mean squared error (MSE), while the vertical line on the right corresponds to the λ value that yields one standard error away from the minimum MSE. The optimal value was determined by selecting the left vertical line for the investigation.

Taking the treatment outcome (effective or ineffective) as the dependent factor and other items as independent factors, a lasso regression analysis was performed on the training data (Figure 2a). According to the results (Figure 2b), five variables were selected when λ = 0.073: age, minHR, rMSSD, SDANN, and HF. Multiple collinearities detected before the logistic regression analysis showed that all VIF were <10 (Table 3). A Spearman correlation matrix revealed the correlation within the five variables selected (Table 4). The aforementioned parameters were filtered on the logistic regression analysis, revealing that age (odds ratio [OR] = 1.347; 95% confidence interval [CI] = 1.04–1.74) and rMSSD (OR = 1.057; 95% CI = 1.02–1.09) were predictors of its effectiveness (Table 5). We performed a linearity assumption check for the two variables, and there was no significant correlation between the two. Thus, the therapeutic efficacy model’s equation is presented below:

logit(p) = −6.088 + 0.298 × age + 0.056 × rMSSD

### 3.3. Visual Predictive Model Construction and Model Evaluation

**Figure 3:**
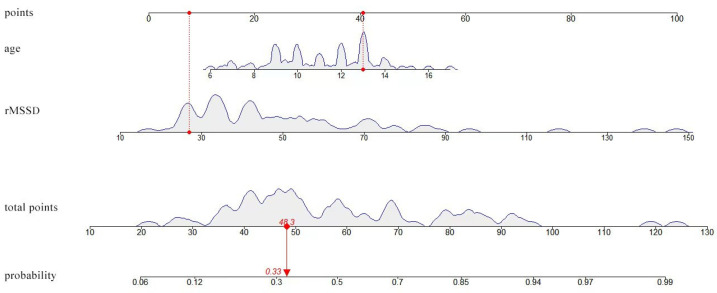
The visual predictive model developed using a nomogram for estimating the therapeutic effect of orthostatic training in children with VVS. rMSSD, the root means square of successive differences between normal sinus beats. For example, for a 13-year-old patient with an rMSSD of 26 ms, the corresponding points (red dots) were acquired by drawing vertical lines through the points representing the values of the two parameters and perpendicular to the axes of the points (red dashed line), resulting in approximately 40 points. The corresponding probability is 0.316 (red double arrow); therefore, orthostatic training is not recommended.

We constructed a visual predictive model using a nomogram based on the prediction model to serve as a classical method for estimating the effectiveness rate of orthostatic training for the individual patients (Figure 3). Scores were assigned to each factor by forming a line with designated points on the axis. The estimation of efficacy can be derived by using the total scores. For clinical application, the sum of the scores is first calculated based on the points corresponding to Age and rMSSD on the “points” line, and this value is “total points”, used to find the corresponding value to the “probability” line, which is the probability that orthostatic training is effective in the patient. Detailed examples can be found in Figure 3.

**Figure 4:**
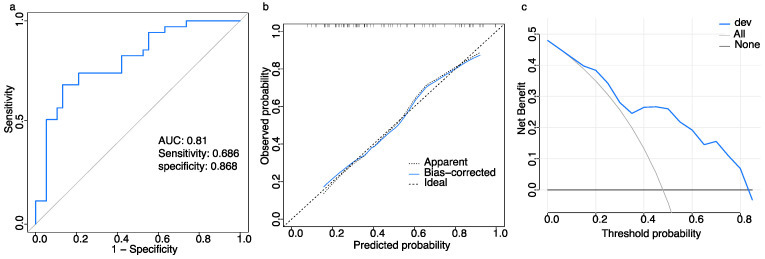
Model evaluation of the training dataset for estimating the therapeutic effect of orthostatic training in children with VVS. (**a**) Receiver operating characteristic (ROC) curves. (**b**) Calibration curve. The x and y axes represent the predicted probabilities and observed probabilities. The line labeled “ideal” is the reference line indicating perfect calibration, whereas the line labeled “apparent” represents the performance of visual predictive model. The intercept is 0.00 (95% CI, −0.54–0.54), slope is 1.00 (95% CI, 0.49–1.51), and c-statistic is 0.81 (95% CI, 0.69–0.89). The bias-corrected line represents the visual predictive model performance corrected by bootstrapping (B = 1000 repetitions). (**c**) Decision curve analysis (DCA). The *y*-axis indicates the net benefit, and the *x*-axis indicates the threshold probability. The line labeled ‘none’ corresponds to the scenario where no patients receive orthostatic training, whereas the line labeled ‘all’ corresponds to the scenario where all patients receive orthostatic training.

The AUC of the ROC curve, which assessed the model’s predictive performance on the training data, was 0.81 (95% CI, 0.71–0.91: Figure 4a). The logit(p) is 0.048 when the Youden index is at its maximum, the specificity was 0.868, and sensitivity was 0.686. The actual and predicted probabilities fit the calibration plots well (Figure 4b). In addition, using the visual predictive model to predict the therapeutic effect demonstrated a greater net benefit over a threshold range of 0.15–0.85 (Figure 4c). For instance, when the threshold was 0.6, the net benefit was approximately 20% in the visual predictive model, which is higher than that of the non-selective treatment.

### 3.4. Model Validation

**Figure 5:**
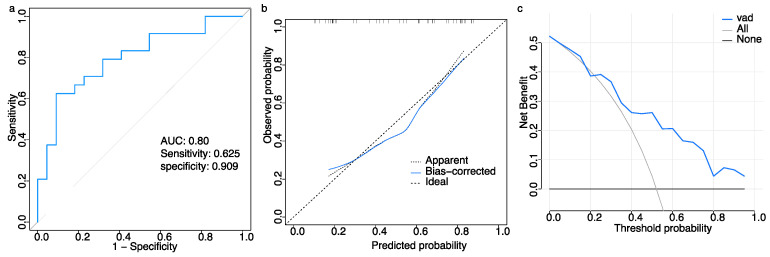
Model evaluation of the validation dataset for estimating the therapeutic effect of orthostatic training in children with VVS. (**a**) Receiver operating characteristic (ROC) curves. (**b**) Calibration curve. The x and y axes represent the predicted probabilities and observed probabilities. The line labeled “ideal” is the reference line indicating perfect calibration, whereas the line labeled “apparent” represents the performance of visual predictive model. The intercept is −0.12 (95% CI, −0.82–0.58), slope is 0.89 (95% CI, 0.32–1.45), and c-statistic is 0.80 (95% CI, 0.63–0.90). The bias-corrected line represents the visual predictive model performance corrected by bootstrapping (B = 1000 repetitions). (**c**) Decision curve analysis (DCA). The *y*-axis indicates the net benefit, and the *x*-axis indicates the threshold probability. The line labeled ‘none’ corresponds to the scenario where no patients receive orthostatic training, whereas the line labeled ‘all’ corresponds to the scenario where all patients receive orthostatic training.

The predictive model was evaluated using validation data. The ROC curve displayed an AUC of 0.80 (95% CI, 0.66–0.93) (Figure 5a), and logit(p) is 0.765 when the Youden index is at its maximum; the sensitivity and specificity for estimating the therapeutic effects of the orthostatic training in the validation dataset were 0.625 and 0.909, respectively (Figure 5b). As shown in Figure 5b, the calibration plots exhibit a good association between the actual and predicted probabilities. In addition, using a visual predictive model to predict the therapeutic efficacy demonstrated a greater net benefit over a threshold range of 0.2–1.0 (Figure 5c).

## 4. Discussion

Autonomic nerve–mediated syncope (NMS) accounts for >70% of syncope cases in children; of NMS types, VVS is the most common [34,35]. However, the prognosis of VVS is not always benign [36]. Therefore, numerous treatment methods, including pharmacological and nonpharmacological therapies, have been applied [37]. Nonpharmacological treatments, such as orthostatic training, comprise one-third of the NMS treatment options for children [35]. Our research investigated the markers associated with the effectiveness of orthostatic training. This study has two key findings: (1) according to the multivariable logistic regression analysis, age and rMSSD were associated with the therapeutic effect; and (2) a novel visual predictive model developed here demonstrated good predictive efficiency and made the clinical application more convenient.

In the comparison of baseline factors, age and HRV parameters differed significantly between the two groups. Orthostatic training was significantly more effective in older children. This phenomenon is believed to be associated with patient compliance. However, additional studies are required to confirm this finding. The SDNN, SDNN Index, rMSSD, SDANN, pNN50, LF, and HF values were higher in the effective versus the ineffective group. Previous research [38] demonstrated that the parameters of HRV can reflect changes in autonomic tone that are mainly influenced by the parasympathetic nerves. Higher parasympathetic nerve activity results in a lower HR. The minHR and meanHR values were lower in the effective versus ineffective treatment group. These results imply that parasympathetic nerve activity increased in the effective group. A randomized placebo-controlled study [6] indicated that daily orthostatic training increased autonomic tone, especially sympathetic activity, which may help balance autonomic function. And LF/HF is lower in the effective group (*p* < 0.05), which is closer to 1.2, the ratio of LF/HF in healthy adults [22], than that in the ineffective group.

A prediction model was constructed using a combination of the least absolute shrinkage and selection operator (lasso) and logistic regression. The lasso regression reduces the regression coefficients to zero, resulting in the selection of significant variables and the enhancement of the interpretability of the model [39]. Compared to the traditional approach, lasso regression has been utilized in several medical domains to predict outcomes, such as depressive disorder [40], parotid tumors [41], arthritis [42], intracerebral hemorrhage [43], diabetes mellitus [44], and emergency triage [45]. Two factors were included in the prediction model: age and rMSSD. Older age was related to a better response to orthostatic training (ORage = 1.347), which meant that the odds of an effective response increased by 34.7% when the patient’s age increased by 1 year in the case of the same rMSSD. We suppose that treatment compliance may be one explanation for these results; however, further research is needed to provide an exact explanation.

The time-domain parameter rMSSD was another index in the prediction model (OR rMSSD = 1.057), indicating that increased rMSSD was related to better therapy outcomes at the same age. The rMSSD value is a well-established metric that quantifies the variability in HR from one beat to the next. It is widely recognized as the principal time-domain measure used to assess vagal activity [46]. The regulation of the cardiac autonomic nervous system, which encompasses the sympathetic nervous system (SNS) and parasympathetic nervous system (PNS), is governed by cardiovascular regulatory centers located inside the brainstem [47] that maintain a relatively dynamic balance between the SNS and PNS in healthy individuals. A previous study revealed that orthostatic training may help balance the SNS and PNS and restore upright tolerance, at least in part, by enhancing peripheral vasoconstriction that can be achieved during continuous upright stimuli [48]. Therefore, an increased rMSSD may suggest a disturbance in the autonomic nervous system at baseline and predict an optimal therapeutic response to orthostatic training.

The minHR, SDANN, and LF were also screened by lasso regression, but were not included in the prediction model, for which we have two conjectures. First, both HR and HRV parameters are age-related [17]. In the training dataset, age is higher in the effective versus ineffective group (*p* < 0.05), the differences in minHR, SDANN, and LF between the two groups may be age-related, resulting in non-inclusion. Second, this is a single-center retrospective study with a small sample size, and there may be selection bias resulting in the failure to enroll certain indicators. Further conclusions should be drawn from a more extensive sample of the target population to minimize the impact of selection bias.

A nomogram is a straightforward visual depiction [49]. By visually displaying the influence of each predictor on an outcome, a more concrete understanding is offered. We developed a visual predictive model using a nomogram in this study. In the training dataset, the prediction model was evaluated using ROC with an AUC of 0.81 and a calibration curve with good calibration. Nevertheless, the use of ROC curves and calibration plots may not adequately capture the clinical implications. The assessment of the applicability of a prediction model in clinical scenarios and the accompanying benefits to patients rely heavily on its clinical usefulness. To showcase the practicality of the model, we conducted an assessment to determine whether judgments aided by the model resulted in enhanced patient outcomes using the DCA method. When comparing the treatment of all versus no patients, model-assisted judgments yielded a higher overall benefit across various thresholds in the training and validation datasets.

There are other methods that can be employed to evaluate the autonomic function and which can be considered to predict the outcome of orthostatic training. According to a review on the assessment of autonomic function from Lu et al., several methods, including bedside cardiac autonomic tests, HUTT, standing test, QT interval dispersion, sympathetic skin responses, and detection of catecholamines, have been applied in this field [50]. Such bedside autonomic function tests as the Valsalva maneuver, deep breathing test, and orthostatic challenges, together with HUTT, all require the child patient to complete certain actions. The reliability of the outcomes may, to a certain extent, depend on the young patient’s cooperation and the standardization of these procedures. Some indexes derived from the electrocardiogram, like QT interval dispersion, can also be used to evaluate the autonomic dysfunction [51]. However, the complicated measurement work may bring some trouble to their applications. The sympathetic skin response test and determination of blood catecholamines may cause discomfort to the child patient. The value of the above indicators in predicting therapeutic response to orthostatic training in VVS patients has yet to be confirmed in clinical trials. A study from Chun et al. revealed that, in adults with a neurally mediated syncope, a baroreflex sensitivity (BRS) < 8.945 ms/mmHg in the supine position was significantly and independently related to the ineffectiveness of tilt training (odds ratio 23.10; 95% CI 1.20–443.59; *p* = 0.037) [52]. The BRSs were computed by the Finapres Medical System during HUTT [52]. The value of BRS in forecasting outcomes of tilt training in children needs further investigation. A previous study of 33 children with VVS showed that the acceleration index can predict the efficient rate of orthostatic training [12]. However, the predictive specificity of the acceleration index was 69.2%, which was not so satisfying.

This research is accompanied by several limitations. First, as a retrospective study, it is difficult to control the compliance of daily orthostatic training, which could have influenced the outcomes to a certain extent. Second, the sample size was small, and subjects were divided into a training and validation dataset based on admission date, which might introduce selection bias leading to exclusion of certain variables. Third, more comprehensive data of HRV should be further analyzed, such as the diurnal and nocturnal data of the time domain as well as frequency domain, in addition to some non-liner indicators. Fourth, this study did not consider other control factors affecting syncope recurrence, such as autonomic dysfunction indices, increasing fluid ingestion, avoiding triggers, improving physical fitness, etc., which may have biased the results. Fifth, as a single-center study, no external validation was performed. In subsequent periods, the data collection continued to evaluate the effectiveness of the model. Sixth, we note that some studies revealed that HRV parameters are age-dependent [17,53], and it will be more ideal to calculate z-scores for all of the parameters and to construct a more precious model. For this purpose, standardized HRV values depending on age as well as other indexes of our studied population are required. We will pay attention to this issue in our future work.

## 5. Conclusions

In summary, this study explores a visual predictive model for predicting the efficient rate of orthostatic training in children with VVS. The results of the training and validation datasets demonstrate that this predictive model is accurate and consistent. The model holds promise for clinicians in quickly determining the probability that a child will benefit from orthostatic training based on the measurement of age and rMSSD, with the advantages of non-invasiveness and convenience.

## Figures and Tables

**Table 1:** Baseline variables of orthostatic training in children with vasovagal syncope.

Variables	Total, N = 119	Training Data, N = 73	Validation Data, N = 46	*p*
Age (years)	12 (9, 13)	11 (9, 13)	12 (9, 14)	0.190
Sex (Male/Female, N)	60/59	36/37	24/22	0.761
BMI (kg/m^2^)	19.1 (16.8, 22.2)	18.4 (16.5, 21.2)	20.8 (17.0, 24.1)	0.094
Total attacks of syncope before treatment (times)	2 (1, 5)	3 (2, 5)	2 (1, 3)	0.016
Therapy duration (months)	3 (3, 3)	3 (3, 3)	3 (2, 3)	0.779
SBP (mmHg)	110 (102, 118)	109 (101, 116)	115 (104, 125)	0.067
DBP (mmHg)	67 ± 7.8	66 ± 7.4	67 ± 8.4	0.414
minHR (bpm)	50 ± 6.9	51 ± 6.7	49 ± 7.1	0.062
maxHR (bpm)	148 (138, 161)	150 (141, 162)	145 (127, 156)	0.067
meanHR (bpm)	82 (74, 90)	84 (78, 91)	81 (70, 90)	0.087
SDNN (ms)	147.9 ± 36.7	140.7 ± 32.1	159.4 ± 40.8	0.006
SDNN Index (ms)	70 (57, 87)	65 (56, 82)	75 (64, 94)	0.007
rMSSD (ms)	45 (34, 60)	42 (33, 60)	48 (39, 62)	0.245
SDANN (ms)	136.0 ± 40.5	132.5 ± 40.1	141.6 ± 40.9	0.237
pNN50 (%)	21 (11, 31)	18 (10, 26)	24 (16, 34)	0.012
LF (ms^2^)	1025 (699, 1335)	1051 (738, 1342)	940 (639, 1372)	0.413
HF (ms^2^)	636 (359, 1034)	718 (401, 1155)	565 (337, 912)	0.230
LF/HF	1.7 (1.2, 2.0)	1.7 (1.2, 2.1)	1.7 (1.3, 2.0)	0.637

BMI, body mass index; DBP, diastolic blood pressure; maxHR: maximal heart rate; mean HR, mean heart rate; minHR, minimum heart rate; pNN50, percent of successive NN intervals that differ by more than 50 ms; SBP, systolic blood pressure; SDANN, standard deviation of the averages normal-to-normal intervals in all 5-min segments; SDNN, standard deviation of normal-to-normal (NN) intervals; rMSSD, the root means square of successive differences between normal sinus beats; SDNN Index, standard deviation of NN interval index; LF, low frequency; HF, high frequency; LF/HF, the LF to HF proportion.

**Table 2:** Comparison of effective and ineffective subgroups in the training dataset.

Variables	Ineffective, N = 38	Effective, N = 35	*p*
Age (years)	10 (9, 12)	12 (10, 13)	0.032
Sex (Male/Female, N/N)	19/19	17/18	0.903
BMI (kg/m^2^)	18.3 (17.0, 21.5)	18.7 (16.3, 21.0)	0.947
Total attacks of syncope before treatment (times)	3 (1, 5)	3 (2, 5)	0.523
Therapy duration (months)	3 (3, 3)	3 (2, 3)	0.585
SBP (mmHg)	110 (101, 116)	108 (100, 121)	0.623
DBP (mmHg)	65 ± 7.3	67 ± 7.4	0.246
minHR (bpm)	54 ± 6.3	48 ± 6.0	<0.001
maxHR (bpm)	150 (138, 157)	150 (142, 166)	0.855
meanHR (bpm)	88 (82, 93)	80 (74, 88)	0.003
SDNN (ms)	129.3 ± 27.0	153.1 ± 32.9	0.001
SDNN Index (ms)	59 (50, 68)	72 (64, 88)	<0.001
rMSSD (ms)	35 (31, 43)	56 (44, 72)	<0.001
SDANN (ms)	118.1 ± 30.1	148.3 ± 43.9	0.001
pNN50 (%)	12 (8, 20)	24 (15, 36)	0.001
LF (ms^2^)	938 (569, 1099)	1194 (837, 1637)	0.005
HF (ms^2^)	522 (326, 838)	1034 (570, 1453)	<0.001
LF/HF	1.7 (1.3, 2.2)	1.3 (0.7, 1.8)	0.007

BMI, body mass index; DBP, diastolic blood pressure; maxHR: maximal heart rate; mean HR, mean heart rate; minHR, minimum heart rate; pNN50, percent of successive NN intervals that differ by more than 50 ms; SBP, systolic blood pressure; SDANN, standard deviation of the averages normal-to-normal intervals in all 5-min segments; SDNN, standard deviation of normal-to-normal (NN) intervals; rMSSD, the root means square of successive differences between normal sinus beats; SDNN Index, standard deviation of NN interval index; LF, low frequency; HF, high frequency; LF/HF, the LF to HF proportion.

**Table 3:** Multiple collinearity analysis before logistic regression analysis.

Variables	Tolerance	VIF
Age (years)	0.645	1.551
minHR (bpm)	0.385	2.598
rMSSD (ms)	0.185	5.392
SDANN (ms)	0.521	1.918
HF (ms^2^)	0.265	3.777

minHR, minimum heart rate; SDANN, standard deviation of the averages normal-to-normal intervals in all 5-min segments; rMSSD, the root means square of successive differences between normal sinus beats; HF, high frequency; VIF, variance inflation factor.

**Table 4:** Spearman correlation analysis of five variables selected.

	r/*p* Value	Age	rMSSD	SDANN	minHR	HF
Age	r	1	0.012	0.214	−0.467	−0.085
	*p*	-	0.921	0.069	<0.001	0.477
rMSSD	r	0.012	1	0.580	−0.679	0.808
	*p*	0.921	-	<0.001	<0.001	<0.001
SDANN	r	0.214	0.580	1	−0.665	0.413
	*p*	0.069	<0.001	-	<0.001	<0.001
minHR	r	−0.467	−0.679	−0.665	1	−0.468
	*p*	<0.001	<0.001	<0.001	-	<0.001
HF	r	−0.085	0.808	0.413	−0.468	1
	*p*	0.477	<0.001	<0.001	<0.001	-

minHR, minimum heart rate; SDANN, standard deviation of the averages normal-to-normal intervals in all 5-min segments; rMSSD, the root means square of successive differences between normal sinus beats; HF, high frequency; r, Spearman correlation coefficient.

**Table 5:** Multivariate logistic regression results.

Variables	B	SE	OR	95%CI	*p*
Age (years)	0.298	0.131	1.347	1.04–1.74	0.023
rMSSD (ms)	0.056	0.017	1.057	1.02–1.09	0.001
Intercept	−6.088	1.755	0.002		0.001

95%CI, 95% confidence interval; OR, odds ratio; rMSSD, the root means square of successive differences between normal sinus beats.

## Data Availability

The original contributions presented in this study are included in the article. Further inquiries can be directed to the corresponding author.
